# Enhanced representation learning with temporal coding in sparsely spiking neural networks

**DOI:** 10.3389/fncom.2023.1250908

**Published:** 2023-11-21

**Authors:** Adrien Fois, Bernard Girau

**Affiliations:** Université de Lorraine, Centre National de la Recherche Scientifique, Laboratoire lorrain de Recherche en Informatique et ses Applications, Nancy, France

**Keywords:** spiking neural networks, temporal code, spike-timing-dependent plasticity, representation learning, visual representations, latency-coding, sparsity, unsupervised learning

## Abstract

Current representation learning methods in Spiking Neural Networks (SNNs) rely on rate-based encoding, resulting in high spike counts, increased energy consumption, and slower information transmission. In contrast, our proposed method, Weight-Temporally Coded Representation Learning (W-TCRL), utilizes temporally coded inputs, leading to lower spike counts and improved efficiency. To address the challenge of extracting representations from a temporal code with low reconstruction error, we introduce a novel Spike-Timing-Dependent Plasticity (STDP) rule. This rule enables stable learning of relative latencies within the synaptic weight distribution and is locally implemented in space and time, making it compatible with neuromorphic processors. We evaluate the performance of W-TCRL on the MNIST and natural image datasets for image reconstruction tasks. Our results demonstrate relative improvements of 53% for MNIST and 75% for natural images in terms of reconstruction error compared to the SNN state of the art. Additionally, our method achieves significantly higher sparsity, up to 900 times greater, when compared to related work. These findings emphasize the efficacy of W-TCRL in leveraging temporal coding for enhanced representation learning in Spiking Neural Networks.

## 1 Introduction

Spiking Neural Networks (SNNs) have been gaining recognition due to their application in supervised (Kheradpisheh and Masquelier, 2020; Lee et al., 2020), reinforcement (Mozafari et al., 2018; Patel et al., 2019), and unsupervised learning tasks (Diehl and Cook, 2015; Kheradpisheh et al., 2018). Compared to Artificial Neural Networks (ANNs) that output continuous values synchronously, SNNs transmit binary outputs sparsely and asynchronously as spikes. This event-based information transmission scheme reduces communication channels, significantly lowers energy requirements, and offers potential energy efficiency gains of up to 1,000 times with neuromorphic processors (Furber, 2016) compared to traditional processors. The massively parallel architecture of neuromorphic processors, where memory (synapses) and computational units (neurons) are co-located, further contributes to this energy efficiency. By utilizing local event-based learning rules, such as Spike-timing-dependent plasticity (STDP), which leverage information from presynaptic and postsynaptic terminals, the principles of parallelism and locality can be fully exploited at the algorithmic level.

Extracting representations from event-based and asynchronous data streams using local rules compatible with neuromorphic processors holds great promise. In this context, King et al. (2013) proposed a spatially local rule learning receptive fields resembling Gabor filters. Burbank (2015) developed a method that reproduces the behavior of an autoencoder with an STDP rule. This STDP rule learns encoding and decoding weights with a Hebbian and anti-Hebbian rule, respectively. The combination of these two rules allows the approximation of the cost function of an autoencoder. More recently, Tavanaei et al. (2018) proposed an STDP rule, integrating a vector quantization module and a regularization module and provides the best performance in terms of reconstruction error.

However, all these methods operate with spike rate encoding, where information is encoded in the number of emitted spikes. Spike rate encoding incurs high energy costs, and leads to slow information transmission. In contrast, temporal codes, which transmit information based on spike times rather than spike rates, offer a more economical alternative. Temporal encoding using relative latencies achieves significantly higher energy efficiency, reduces the number of spikes required, and enables faster data transmission rates compared to rate coding. This makes temporal codes particularly relevant for neuromorphic computing and learning (Guo et al., 2021).

In this paper, we introduce a two-layer SNN that leverages temporal codes, specifically population-based latency coding, where each neuron fires a single spike. Our Weight-Temporally Coded Representation Learning (W-TCRL) model improves the reconstruction performance while increasing the sparsity of the activity in the network. We propose a novel STDP rule that adjusts synaptic weights based on spike times, operating locally in both space and time, thus facilitating a future implementation on neuromorphic processors (Davies et al., 2018). To the best of our knowledge, this is the first presented method for learning representations from a temporal encoding of inputs using an STDP-like rule aimed at achieving low reconstruction error. We evaluate our spiking model using the MNIST dataset and a natural image dataset.^1^ Furthermore, we propose a generic parameterization of the model to address the common adaptability issue with data of varying dimensions.

## 2 Materials and methods

In this section, the various architectural and algorithmic components of the neural model are illustrated, which will be utilized for representation learning. The model is assessed on an image reconstruction task, with a focus on its new STDP rule that targets synaptic weights.

### 2.1 Spiking neural network

**Figure 1:**
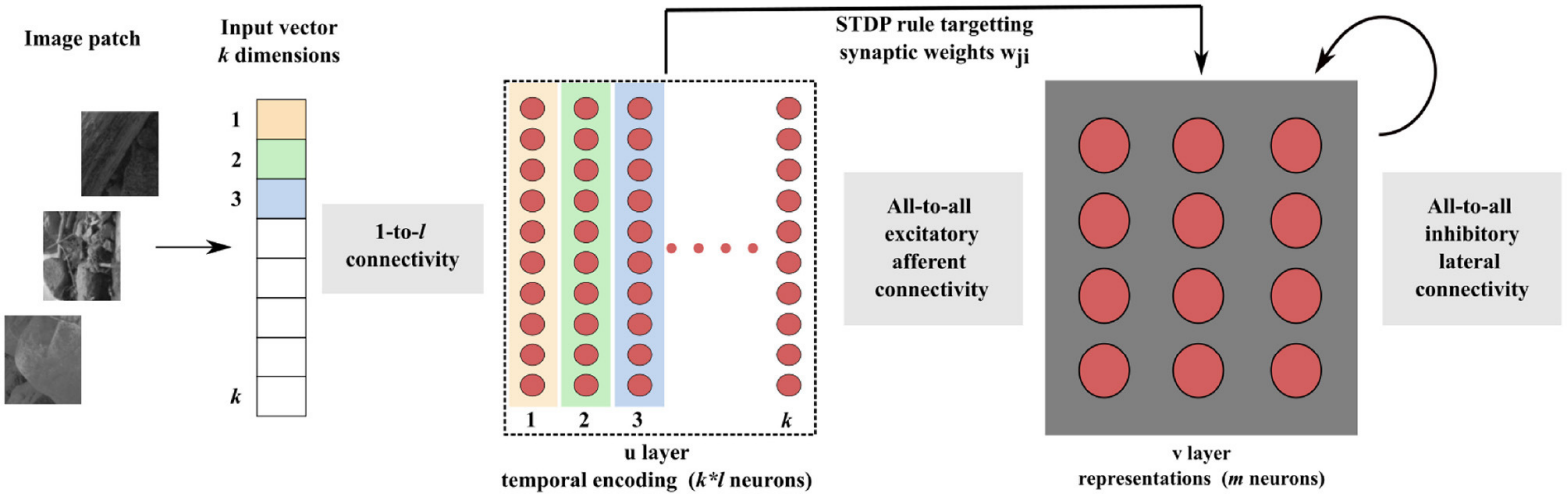
Block diagram of the SNN architecture. Consider an input vector of *k* dimensions. Each dimension of the input vector is encoded by *l* neurons, each of them firing a single spike. The sparse activity of the *k* * *l* neurons is transmitted to the representation layer. Learning of the synaptic weights *w*_*ji*_ takes place between these two layers, with our new STDP rule. Inhibitory lateral connections are present in the representation layer to put neurons into competition, thereby generating competitive learning.

As depicted in Figure 1, our SNN architecture consists of two fully interconnected layers: the first layer encodes input data into relative spike latencies, generating a single spike per neuron, while the second layer extracts representations from the resulting spiking activity.

#### 2.1.1 Neuron and synapse model

The network contains only LIF neurons with current-based (CuBa) synapses. This widely-used model captures the basic behavior of a biological neuron while maintaining low computational cost and ease of analysis. The dynamic of a LIF neuron is given by:


$$\begin{array}{ll} \tau_{m}\frac{dV\left(t\right)}{dt} & =-V\left(t\right)+I\left(t\right)+g\eta \left(t\right) \end{array}$$

Neurons face a Gaussian white noise process η, scaled by g; g = 0 (no noise) for most tests. However, we test the robustness of the network on MNIST dataset in Section 3.3 by varying g. We add the output *s*(*t*) and the resetting of the membrane potential *V*(*t*) when the firing threshold *V*_θ_ is reached, *V*(*t*) is then maintained at 0 during a refractory period *T*_refrac_:


$$\begin{array}{ll} \text{if }V(t)<V_{\theta }, & \text{then }s(t)=0\\ \text{if }V(t)\geq V_{\theta }, & \text{then }\{\begin{array}{l} s(t)=1\\ V(u)=0\;\forall u\in ]t,t+T_{\text{refrac}}] \end{array} \end{array}$$

Between the two layers, synaptic transmission is modeled by an exponential synapse model. If at least one presynaptic neuron *i* fires, the indicator function *s*_*i*_(*t*) takes the value 1 (0 otherwise) and the input to the postsynaptic neuron *I*_*j*_(*t*) changes instantaneously by an amount equal to the synaptic weight *w*_*ji*_:


$$\begin{array}{ll} I_{j}\left(t\right) & \leftarrow I_{j}\left(t\right)+\overset{n}{\underset{i=1}{\sum }}w_{ji}s_{i}\left(t\right) \end{array}$$

otherwise *I*_*j*_(*t*) exponentially decays over time with a time constant τ_*f*_:


$$\begin{array}{ll} \tau_{f}\frac{dI_{j}\left(t\right)}{dt} & =-I_{j}\left(t\right) \end{array}$$

#### 2.1.2 Synaptic traces

Synapses connect the *n* presynaptic neurons of the encoding layer to the *m* postsynaptic neurons of the representation layer. Each synapse has access to a local state variable *x*_*i*_(*t*) (with *i* = 1, 2, …*n*) that tracks recent presynaptic spikes. This variable is known as presynaptic trace. It is commonly used for efficient and local implementations of STDP rules, both in simulations (Pfister and Gerstner, 2006) and neuromorphic processors (Davies et al., 2018).

Similarly, a postsynaptic trace *y*_*j*_(*t*) (with *j* = 1, 2, …*m*) accessible to the synapses tracks recent postsynaptic spikes. When a pre (post)-synaptic spike occurs, *x*_*i*_(*t*) (*y*_*j*_(*t*)) jumps to 1 and then decays exponentially to 0 with a time constant τ_*x*_ (τ_*y*_):


(1) $$\begin{array}{cc} x_{i}(t)\leftarrow 1, & \text{if }s_{i}(t)=\text{1}\\ \tau_{x}\frac{dx_{i}(t)}{dt}=-x_{i}(t), & \text{otherwise} \end{array}$$

(2) $$\begin{array}{cc} y_{j}(t)\leftarrow 1, & \text{if }s_{j}(t)=\text{1}\\ \tau_{y}\frac{dy_{j}(t)}{dt}=-y_{j}(t), & \text{otherwise} \end{array}$$

where *s*(*t*) is an indicator function returning 1 when a neuron fires a spike at time *t*, 0 otherwise.

### 2.2 Encoding input data in relative spike latencies

**Figure 2:**
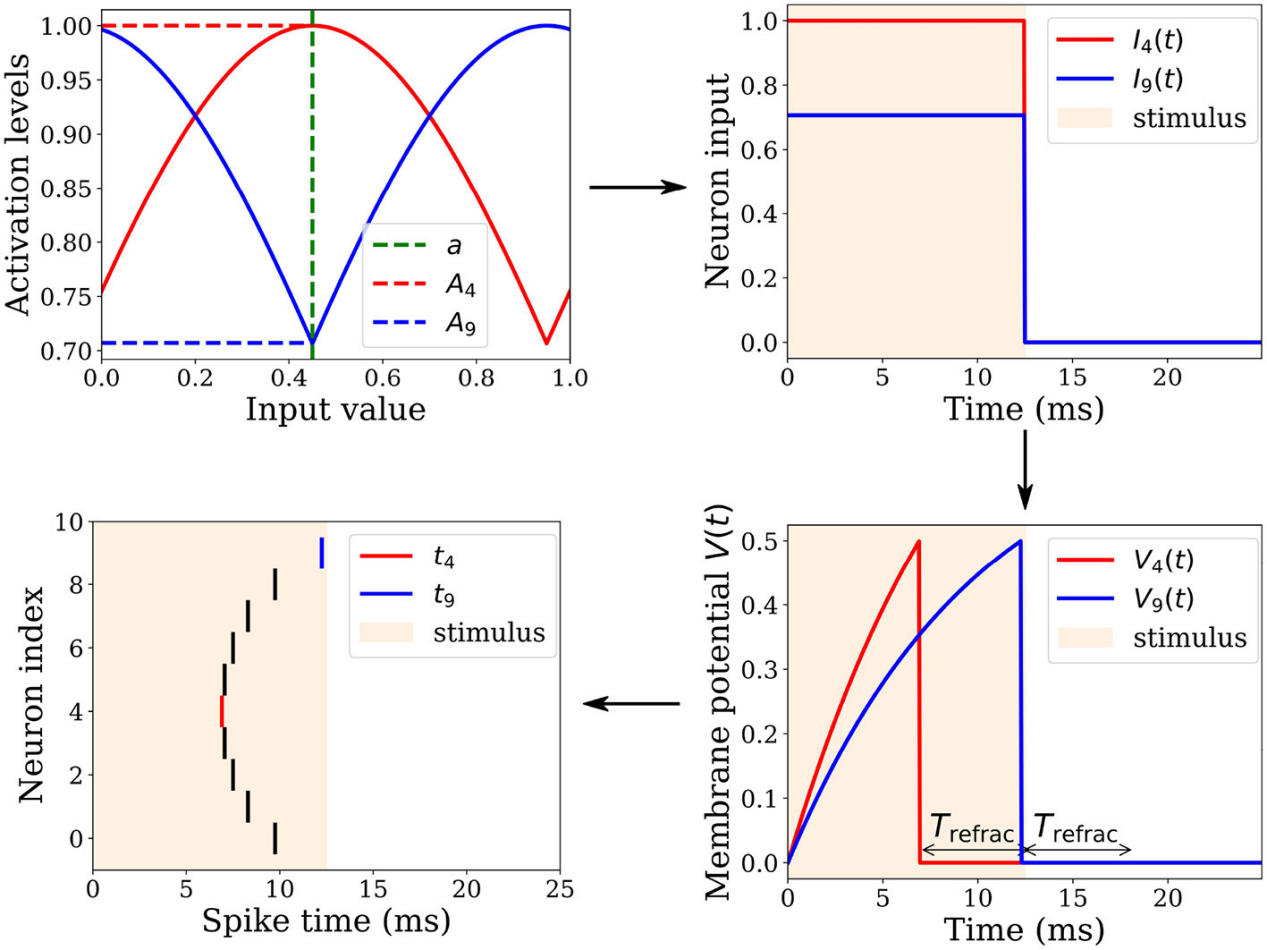
Spatio-temporal spike pattern encoding an input value of *a* = 0.45. **Top left**: The input is received by a population of *l* = 10 neurons, each having a Gaussian tuning curve centered on a preferential value *μ*_*i*_. For instance, Neuron 4 and 9 possess *μ*_4_ = 0.45 and *μ*_9_ = 0.95, respectively. Their tuning curves and corresponding activation levels *A*_4_ and *A*_9_ for the input value are depicted in red and blue, respectively. **Top right**: These activation levels remain constant over a 12.5 ms interval, followed by an equal duration of quiescence. **Bottom right**: The neuronal inputs are integrated into membrane potentials *V*_4_ and *V*_9_. With a higher activation level, *V*_4_ grows faster than *V*_9_, enabling it to reach its firing threshold *V_θ_* sooner. Upon crossing this threshold, the neuron fires a spike, resets its membrane potential, and enters a refractory period *T*_refrac_. **Bottom left**: A neuron fires a spike when it crosses its threshold *V_θ_*, thereby distributing an input value across a neuronal population to produce a specific spatio-temporal spike pattern.

Let **a** ∈ [0, 1]^*k*^ denote a normalized *k*-dimensional real-valued input vector that we aim to encode into relative spike latencies (or timing) through population coding (Ebitz and Hayden, 2021). This can be achieved by distributing each dimension of the input vector over the population activity of *l* neurons (Figure 2). We used a population of *l* = 10 neurons to collectively represent one dimension. Each neuron *i* ∈ {1, 2, …, *l*} of the population has an associated gaussian receptive field in a circular space in range [0, 1], characterized by its preferential value (or center) μ_*i*_ and by its width (or standard deviation) σ. The centers μ_*i*_ are uniformly spread between 0.05 and 0.95. The standard deviation σ = 0.6 is the same for all neurons. This broad tuning ensures that any neuron receives high enough activation levels to fire in response to any input value. The resulting receptive fields overlap, covering the entire input space.

A gaussian function *G*_***μ***, *σ*_:**a**→**A** is then used to transform the input vector **a** ∈ [0, 1]^*k*^ into a vector of activations levels **A** ∈ [0, 1]^*k*×*l*^ that feed the *n* = *k* × *l* LIF neurons of the encoding layer.

To illustrate the encoding process, consider a single entry of the input vector *a* ∈ **a**. The *i*th neuron—with *i* ∈ {1, 2, …, *l*}—whose gaussian receptive field center μ_*i*_ is the closest to the input value *a*, gets the highest activation level *A*_*i*_ and will thus fire first. The other neurons fire later. The higher the distance between their centers and the input value, the lower the activation levels they get, and therefore the later they fire. The temporal separation of spikes is accentuated by the non-linear integration of the received activation levels by LIF neurons. With this mechanism, a specific input value is encoded in a specific spike pattern in the spatio-temporal domain. This population-based coding does not lie within the times to first spikes, but explicitly within the relative latencies of the spikes emitted by each neuron.

Note that in the scenario of continuous input presentation, this circuit will disrupt the temporal representation of the analog inputs. Inputs need to be somewhat reset to achieve spike time reproducibility. However, it is straightforward to replace this circuit with others such as the one proposed by Rumbell et al. (2014). This method enables continuous input presentation to the network by producing oscillatory firing through an inhibitory mechanism. Thus, it generates an internal time reference through oscillations. Both encoding methods utilize a population of Gaussian receptive fields and yield identical spiking patterns. Rumbell's method offers greater flexibility at the cost of a more complex encoding circuit.

### 2.3 W-TCRL: STDP rule for learning representations in weights

Non-weight-dependent Spike-Timing-Dependent Plasticity (NSTDP) rules can produce stable receptive fields; however, they are bistable, leading to synaptic weights saturating at minimum and maximum values (Song et al., 2000). Hence, the potential range of learning parameters, the synaptic weights in this case, is not fully utilized under NSTDP rules. This limitation significantly impedes the expressive capacity of synaptic weights, diminishing their capability to finely represent external states, such as environmental states.

On the other hand, Weight-dependent Spike-Timing-Dependent Plasticity (WSTDP) rules rectify this issue by generating unimodal and continuous distributions of synaptic weights, thereby leveraging the entire range of weight variation. However, these rules fall short in generating stable receptive fields (Billings and van Rossum, 2009).

Our STDP rule provides the dual advantages of stability and the complete utilization of the synaptic weight range for representation storage. The interplay between our STDP rule and the unimodal temporal code results in a stable and unimodal distribution of synaptic weights (see Figure 4). Here, the magnitude of a synaptic weight is function of the temporal distance between a presynaptic and a postsynaptic spike. In the encoding layer *u*, faster firing neurons carry a more accurate representation of the input, correspondingly leading to a higher value of the synaptic weight. This, in turn, boosts the neuron's causal influence over the emission of a postsynaptic spike.

Our novel STDP rule, derived from a vector quantization criterion, operates both spatially and temporally. Spatially, it focuses on a single pair of presynaptic and postsynaptic neurons *i* and *j*, and temporally, it operates within a defined temporal window.

**Figure 3:**
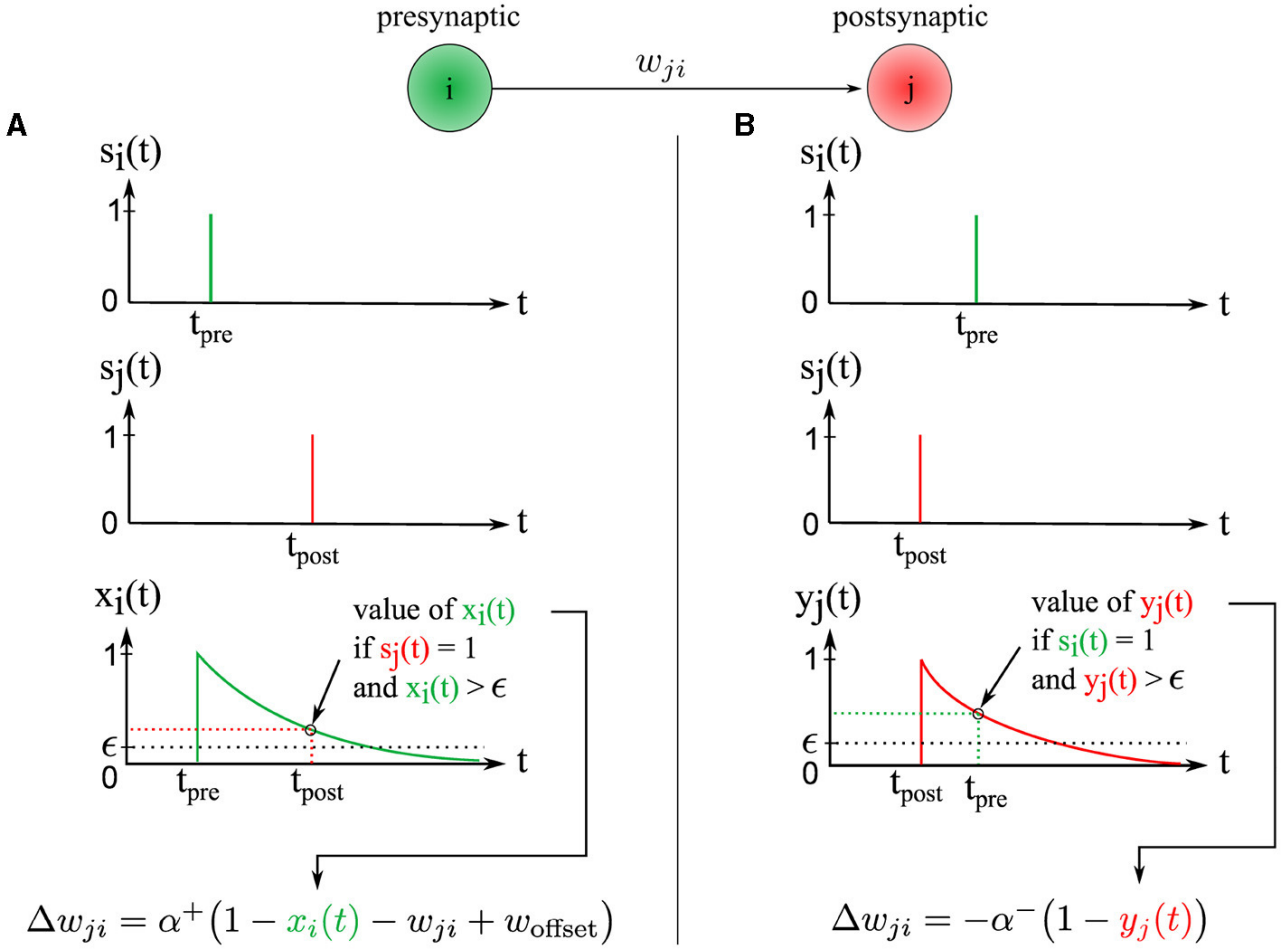
Illustration of the two synaptic weight adaptation cases under our novel STDP rule, demonstrating spatial and temporal locality. **(A)** The left column presents the first case, triggered in a causal context where the presynaptic spike precedes the postsynaptic one. **(B)** Conversely, the right column illustrates the second case, triggered in an anti-causal context when the postsynaptic spike precedes the presynaptic one. In both adaptation cases, the adjustment magnitude Δ*w*_*ji*_ is influenced by the presynaptic and postsynaptic traces *x*_*i*_(*t*) and *y*_*j*_(*t*), respectively. These traces are equivalent to exponential decay kernels applied to Δ_*t*_, where Δ_*t*_ = *t*_post_−*t*_pre_.

The change in synaptic weight Δ*w*_*ji*_ is calculated based on the presynaptic trace *x*_*i*_(*t*) ∈ ]0, 1] and the postsynaptic trace *y*_*j*_(*t*) ∈ ]0, 1]. The synaptic weight *w*_*ji*_ ∈ [0, 1] is then updated by this change *w*_*ji*_←*w*_*ji*_+Δ*w*_*ji*_. Our new STDP rule is defined as follows (see Figure 3):


(3) $$\begin{array}{l} \Delta w_{ji}=\{\begin{array}{ll} +\alpha^{+}(1-x_{i}(t)-w_{ji}+w_{\text{offset}}), & \text{if }s_{j}(t)={\text{1 and }x}_{i}(t)>\epsilon \\ -\alpha^{-}(1-y_{j}(t)), & \text{if }s_{i}(t)=\text{1 and }y_{j}(t)>\epsilon \end{array} \end{array}$$

where α^+^ and α^−^ are the learning rates. This STDP rule works in combination with the introduction of hard bounds that restrict synaptic weights to the range 0 ≤ *w*_*ji*_ ≤ 1:


$$w_{ji}\leftarrow \min(1,\max(0,w_{ji}+\Delta w_{ji}))$$

#### 2.3.1 First case of synaptic weight adaptation

The first adaptation case (Figure 3A) is (1) triggered event-wise by a postsynaptic spike indicated by *s*_*j*_(*t* = *t*_post_) = 1 and (2) operates locally in time since the presynaptic trace *x*_*i*_(*t*) is sampled at time *t* = *t*_post_ and must be greater than ϵ, corresponding to a temporal window since the time of the presynaptic spike *t*_pre_. Thus, postsynaptic spikes arriving outside this temporal window do not trigger the rule. This rule addresses the case of causal interactions, i.e., presynaptic spikes emitted before or at the time of the postsynaptic spike, i.e., Δ_*t*_≥0.

This first adaptation case integrates a vector quantization module aimed at learning the distribution of relative spike times in the distribution of synaptic weights, and thus minimizing the reconstruction error because the neural encoding is temporal. We can observe, by analyzing the equilibrium solution Δ_*w*_*ji*__ = 0, that the weight *w*_*ji*_ converges to a value dependent on *x*_*i*_(*t*) and thus on the time interval Δ_*t*_ = *t*_post_−*t*_pre_ between pre- and postsynaptic spikes:


$$\begin{array}{ll} \Delta w_{ji} & =0\\ \Leftrightarrow \;0 & =1-x_{i}\left(t\right)-w_{ji}+w_{\text{offset}}\\ \Leftrightarrow \;w_{ji} & =1-x_{i}\left(t\right)+w_{\text{offset}}\\ \Leftrightarrow \;w_{ji} & =1-\exp\left(-\frac{t-t_{\text{pre}}}{\tau_{x}}\right)+w_{\text{offset}} \end{array}$$

As this adaptation case is triggered by a postsynaptic spike indicated by *s*_*j*_(*t* = *t*_post_) = 1, we finally obtain:


(4) $$\begin{array}{ll} \Leftrightarrow \;w_{ji} & =1-\exp\left(-\frac{\Delta_{t}}{\tau_{x}}\right)+w_{\text{offset}} \end{array}$$

**Figure 4:**
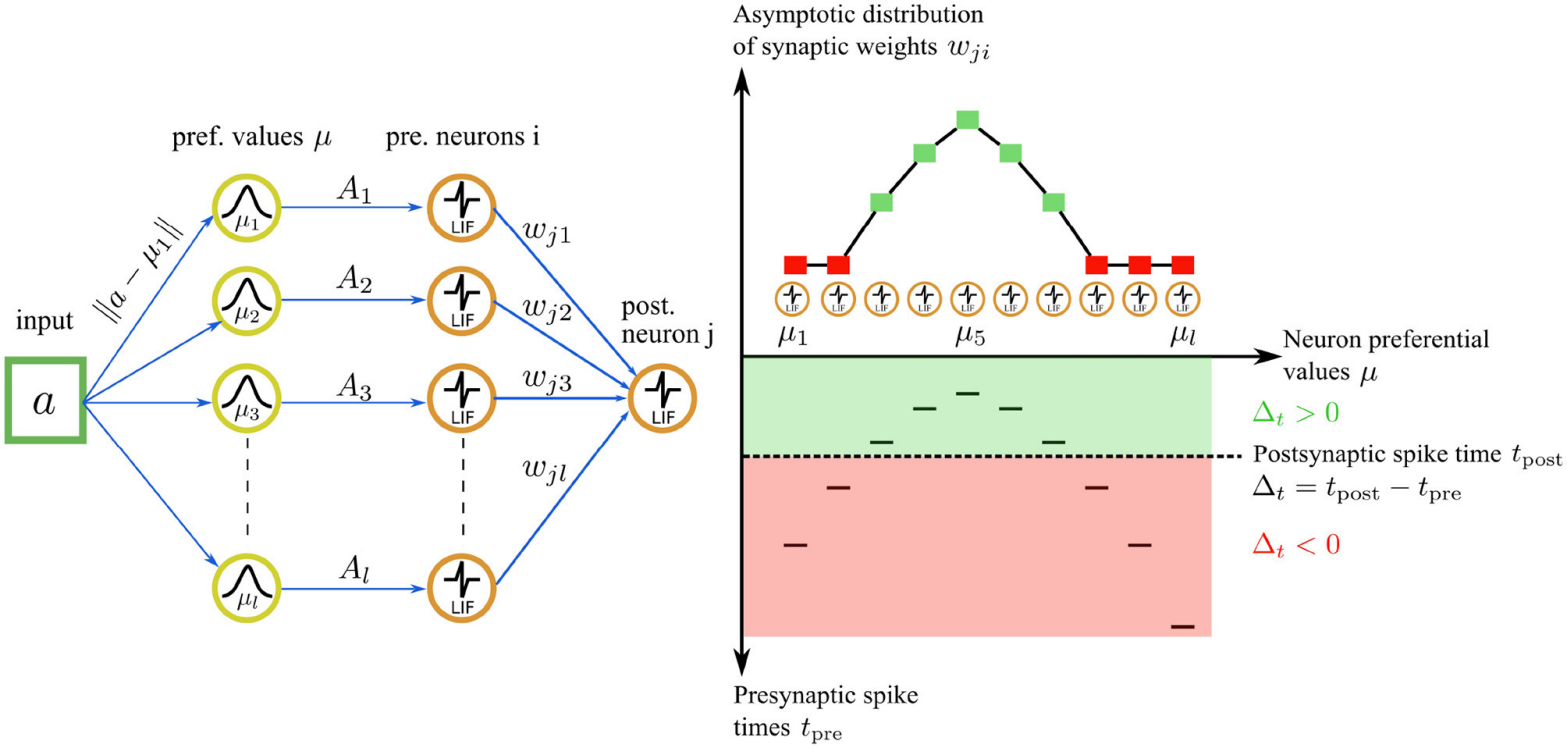
Illustration of the asymptotic weight distribution learned with our STDP rule for an input value of *a* = 0.45. **Left**: The input value is encoded by a population of *l* = 10 neurons in the encoding layer *u*. **Right bottom**: The neuron with the nearest preferential value *μ*_*i*_ to the input (*μ*_5_ = 0.45 in this case) fires a spike first, followed by the other neurons in the population. The spiking pattern is transmitted through synaptic connections to a postsynaptic neuron *j* in the representation layer. The postsynaptic neuron *j* integrates the received inputs and fires a spike at time *t*_post_ (dashed line). The sign of the time difference Δ_*t*_ between a postsynaptic and a presynaptic spike delimits two learning regimes (green and red areas) for the STDP rule. **Right top**: for Δ_*t*_ ≥ 0, the first adaptation case is triggered, resulting in an attractive fixed point weight. The asymptotic weight value *w*_*ji*_ increases as Δ_*t*_ increases (green squares). For Δ_*t*_ ≤ 0, the second adaptation case is triggered, leading to weight depression. The weight value *w*_*ji*_ decreases as Δ_*t*_ increases, tending asymptotically toward 0 (red squares).

Consider a spike pattern repeatedly presented to a postsynaptic neuron for illustration. The synaptic weight *w*_*ji*_ will converge to a value that depends on the presynaptic trace *x*_*i*_(*t*) and therefore on Δ_*t*_. Recall that the trace *x*_*i*_(*t*) jumps to 1 upon the emission of a presynaptic spike, and then decays exponentially over time. Thus, the larger the value of Δ_*t*_ → ∞, the smaller the value of *x*_*i*_(*t*) → 0. The first presynaptic spikes carry most of the information about the input, which can be interpreted as a hidden variable. These first spikes are reflected by a large Δ_*t*_ and thus a small *x*_*i*_(*t*) ∈ ]0, 1]. Therefore we introduce the term 1−*x*_*i*_(*t*) in the rule, which induces high synaptic weight values *w*_*ji*_ for the earliest firing presynaptic neurons, thereby amplifying the causal impact of the first presynaptic spikes on the postsynaptic neuron. Consequently, the first presynaptic neuron that fires induces the highest weight value *w*_*ji*_, while the last firing neuron induces the lowest weight value *w*_*ji*_. This process unfolds for each presynaptic-postsynaptic neuron pair meeting the conditions of the first adaptation case. Thus, the order and latency of the spikes are represented in the resulting synaptic weight distribution *w*_*ji*_ (see Figure 4). By contrast, the traditional STDP rule (Song et al., 2000) maximally strengthens the latest spike (*t*_*pre*_≈*t*_*post*_) for causal interactions and typically leads to weight saturation, where all weights saturate to 1.0 for instance.

We still need to address the role of the last term *w*_offset_. When Δ_*t*_ → 0, this is reflected by *x*_*i*_(*t*) → 1 and induces 1−*x*_*i*_(*t*) → 0 and thus *w*_*ji*_ → 0. To avoid *w*_*ji*_ → 0 when a presynaptic spike is emitted at a time *t*_pre_≈*t*_post_, i.e., when Δ_*t*_ → 0, we add a positive offset *w*_offset_>0 to the rule.

#### 2.3.2 Second case of synaptic weight adaptation

The second adaptation case (Figure 3B) is triggered by a presynaptic spike indicated by *s*_*i*_(*t* = *t*_pre_) = 1, and operates locally in time because the postsynaptic trace *y*_*j*_(*t*) is sampled at time *t* = *t*_pre_ and must be greater than ϵ, corresponding to a temporal window from the postsynaptic spike time *t*_post_. Thus, presynaptic spikes falling outside of this temporal window do not trigger the rule. This rule handles the case of anti-causal interactions, i.e., presynaptic spikes emitted after or at the time of the postsynaptic spike, i.e., Δ_*t*_ ≤ 0.

We can reformulate this adaptation case to explicitly show the dependence on Δ_*t*_ = *t*_post_−*t*_pre_:


$$\begin{array}{ll} \Delta w_{ji} & \propto 1-y_{j}\left(t\right)\\ \Delta w_{ji} & \propto 1-\exp\left(-\frac{t-t_{\text{post}}}{\tau_{y}}\right) \end{array}$$

Since this adaptation case is triggered by a presynaptic spike indicated by *s*_*i*_(*t* = *t*_pre_) = 1, we have:


$$\begin{array}{ll} \Delta w_{ji} & \propto 1-\exp\left(-\frac{t_{\text{pre}}-t_{\text{post}}}{\tau_{y}}\right)\\ \Delta w_{ji} & \propto 1-\exp\left(+\frac{\Delta_{t}}{\tau_{y}}\right) \end{array}$$

Δ*w*_*ji*_ is proportional to $1-\exp(\frac{\Delta_{t}}{\tau_{y}})$, which is multiplied by −α^−^. Therefore, this second adaptation case depresses the synaptic weights. The postsynaptic trace *y*_*j*_(*t*) behaves similarly to the presynaptic trace *x*_*i*_(*t*), where it jumps to 1 upon emission of a postsynaptic spike and then decays exponentially over time. Thus, the larger the value of Δ_*t*_ → ∞, the smaller the value of *y*_*j*_(*t*) → 0. Late-arriving presynaptic spikes convey minimal information about the input. That is why we introduce Δ*w*_*ji*_∝1−*y*_*j*_(*t*) into the rule: the later a presynaptic spike arrives relative to a postsynaptic spike, the greater the depression of *w*_*ji*_. Thus, considering the asymptotic behavior of this synaptic depression case, the weight *w*_*ij*_ → 0 (see Figure 4), as the lower bound of the weight is fixed at 0 using a hard bound. This asymptotical behavior mirrors the traditional STDP rule (Song et al., 2000) for anti-causal interactions. However, the learning dynamics are reversed : the latest spike (*t*_*pre*_≫*t*_*post*_) induces maximal weight depression, while close anti-causal interactions (*t*_*pre*_≈*t*_*post*_) result in minimal weight depression.

#### 2.3.3 Winner-Take-All circuit

Through afferent synaptic weight adjustments, postsynaptic neurons progressively adapt to distinct spatiotemporal patterns (see Figure 4). Additionally, akin to biological neurons, they emit spikes before receiving all presynaptic inputs, thus boosting the SNN's processing speed.

Remember that the first spikes reduce uncertainty about the input the most, which can be interpreted as a hidden variable. This feature is harnessed by a temporal Winner-Take-All circuit. In this circuit, the first neuron crossing its firing threshold *V*_*j*_(*t*)>*V*_θ_ in response to a spatiotemporal input pattern is identified as the best pattern representative, termed the Spiking Best Matching Unit (SBMU). The SBMU strongly inhibits the other neurons in the representation layer, preventing them from firing and thus learning the current prototype.

This self-organizing competitive learning scheme is implemented by all-to-all lateral inhibitory connections. The circuit induces highly sparse activity in the representation layer and forces neurons to learn uncorrelated code vectors, two important ingredients for efficient learning (Bengio et al., 2013; Falez et al., 2019).

However, for a given input vector, a pure WTA circuit only updates the code vector of a single neuron: the SBMU. Our aim is to speed up learning by recruiting more than one neuron (k-WTA) during the presentation of an input vector.

To achieve this goal, we introduce a homeostatic mechanism that increases the value of the lateral synaptic weights in the representation layer. Initially, lateral inhibition is minimal with lateral weights initialized at *w*_*ji*_ = −*c*_*min*_. As learning progresses, the homeostatic mechanism raises these weights toward the target value of *w*_*ji*_≈−*c*_*max*_. With appropriate values of *c*_min_ and *c*_max_, the circuit can dynamically transition from a k-WTA to a WTA behavior.

The homeostatic mechanism progressively involves fewer neurons during the learning phase, enabling gradual decorrelation and specialization of the code vectors that neurons learn. The mechanism is described by the following equation:


(5) $$\begin{array}{ll} \tau_{w}\frac{dw_{ji}}{dt} & =-c_{\text{max}}-w_{ji} \end{array}$$

where τ_*w*_ is the time constant of the homeostatic mechanism. We set τ_*w*_ to one-third of the total learning phase duration to ensure *w*_*ji*_≈−*c*_*max*_ is reached by the end of the learning phase. Given an encoding time window of *T* and *P* input vectors, the time constant is then given by τ_*w*_ = 1/3 × *T*×*P*.

## 3 Experimental protocol and results

### 3.1 Metrics for performance evaluation

We report experiments of representation learning with visual data in this section. A first natural objective is to evaluate the quality of visual representations learned by neurons in the representation layer *v*. For this, we use the Root Mean Square (RMS) reconstruction error between an input vector and the code vector of the associated SBMU, comparing the original and reconstructed image patches.

The Structural Similarity Index Measure (SSIM) is another measure that gauges the structural similarity between two images, rather than pixel-by-pixel difference as done by the Peak Signal-to-Noise Ratio (PSNR) measure based on RMS error. However, SSIM is not used in works related to ours, making it difficult to compare our performances. Furthermore, studies (Horé and Ziou, 2010; Dosselmann and Yang, 2011) have revealed analytical and statistical links between PSNR (based on RMS) and SSIM, indicating that their differences essentially stem from their sensitivity to image degradation. More generally, there is currently no satisfactory visual quality measure that fully captures human perception. Hence, in addition to the RMS reconstruction error used in our quantitative analysis, we provide a qualitative evaluation that visually examines the learned representations and the resulting reconstructed images.

Related works (Burbank, 2015; Tavanaei et al., 2018) use a correlation coefficient-based similarity measure for evaluating visual representation quality. However, we forgo this measure due to its high sensitivity to outliers (Jenkin et al., 1991; Yen and Johnston, 1996), interpretational challenges (Lee Rodgers and Nicewander, 1988), and technical limitations, such as undefined coefficients for image patches with uniform intensity—a common occurrence in the MNIST database, particularly with pure white patches.

Beyond the quality of learned representations, we also evaluate additional SNN characteristics, such as the sparsity of activity in the representation layer and the Euclidean incoherence in the self-organized election process of the SBMU—a new measure that we introduce subsequently. The RMS reconstruction error, sparsity, and incoherence provide insights into the accuracy, efficiency, and self-organization capability of the SNN.

#### 3.1.1 Mean squared error

We use the Root Mean Squared (RMS) error to quantify the difference between an input image patch **a**_*p*_ and a reconstructed patch â_*p*_ (6):


(6) $$\begin{array}{ll} RMS & =\frac{1}{P}\overset{P}{\underset{p=1}{\sum }}\sqrt{\frac{1}{k}\overset{k}{\underset{i=1}{\sum }}{\left(a_{i,p}-{â}_{i,p}\right)}^{2}} \end{array}$$

Here, *k* is the number of pixels in a patch and *P* is the number of patches, and *a*_*i, p*_ is the *i*th pixel of *a*_*p*_.

Recall that each dimension *i* ∈ 1, 2, …, *k* of an input patch is distributed among *l* encoding neurons in the encoding layer. Each of the *z* ∈ 1, 2, …, *l* encoding neurons has an associated preferential value μ_*z*_ ∈ μ_1_, μ_2_, …, μ_*l*_. The encoding layer is fully connected to the representation layer in an all-to-all relationship. Each postsynaptic neuron in the representation layer is thus connected to k*l presynaptic neurons in the encoding layer.

**Figure 5:**
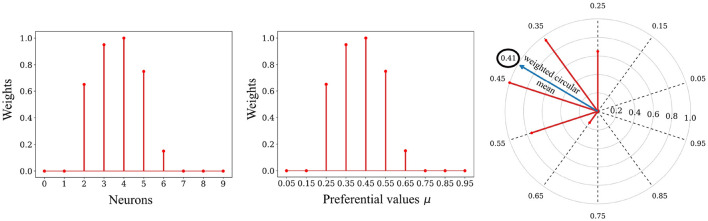
Decoding process. **Left**: The relative spike timing relationships of the spike patterns are stored in the weights. **Middle**: We can replace the index of a neuron with its associated preferential value *μ*. **Right**: The decoding process involves using a circular mean of the encoding neuron's preferential values, weighted by the weights values, to map the stored representation back to the input space. In this example the value 0.41 was decoded from the weights.

Each dimension *i* ∈ 1, 2, …, *k* of the reconstructed patch â_*i, p*_—corresponding in our case to the intensity of a pixel—is decoded from the distribution of *l* synaptic weights $w_{1}^{i},w_{2}^{i},...,w_{l}^{i}$ associated with it. The decoding method is based on the circular mean of neurons' preferred directions, weighted by their weight values, as exposed below (see Figure 5). The underlying principle is that the neural code exhibits unimodality (convexity) due to the presence of circular Gaussian receptive fields. This distribution of spike timings is mirrored in a unimodal synaptic weight distribution in the learned weights, a result of our STDP rule (see Figure 4). Therefore, we employed a circular weighted mean to decode the empirical mean. Interestingly, this decoding method has been successfully employed to decode the direction of arm movement from neuronal populations activity in the primate motor cortex (Georgopoulos et al., 1986).

The first step of the decoding process is to transform each preferential value μ_*z*_ ∈ μ_1_, μ_2_, …, μ_*l*_ of the presynaptic neurons into an angle θ_*z*_ (preferred direction):


$$\begin{array}{l} \theta_{z}=2\pi \mu_{z} \end{array}$$

We then calculate the weighted mean of the Cartesian coordinates ${\bar{x}}_{i}$ and ${\bar{y}}_{i}$ from the angles θ_*z*_ (angular coordinates) and the synaptic weights $w_{z}^{i}$ associated with pixel *i* of the SBMU (radial coordinates).


$$\begin{array}{ll} \text{with }w_{z}^{i} & =w_{\text{sbmu},\left(i-1\right)l+z}\\ \bar{x_{i}} & ={\frac{1}{\Delta }}_{i}\overset{l}{\underset{z=1}{\sum }}w_{z}^{i}\cos\left(\theta_{z}\right)\\ \bar{y_{i}} & ={\frac{1}{\Delta }}_{i}\overset{l}{\underset{z=1}{\sum }}w_{z}^{i}\sin\left(\theta_{z}\right)\\ \Delta_{i} & =\overset{l}{\underset{z=1}{\sum }}w_{z}^{i} \end{array}$$

We calculate a new angle $\bar{\theta_{i}}$ from the Cartesian coordinates ${\bar{x}}_{i}$ and ${\bar{y}}_{i}$. This angle $\bar{\theta_{i}}$ corresponds to the weighted circular mean:


$$\begin{array}{ll} \bar{\theta_{i}} & =atan2\left(-\bar{y_{i}},-\bar{x_{i}}\right)+\pi \end{array}$$

We end with an inverse transformation, transforming the angle $\bar{\theta_{i}}\in \left[0,2\pi \right]$ into the normalized intensity of a pixel in the reconstructed patch â_*i, p*_ ∈ [0, 1] :


$$\begin{array}{ll} {â}_{i,p} & =\frac{\bar{\theta_{i}}}{2\pi } \end{array}$$

#### 3.1.2 Sparsity

Sparsity is not evaluated on the encoding layer but on the output layer, i.e., the representation layer. Sparsity corresponds to the percentage of active neurons for an input vector during the encoding time window *T*:


(7) $$\begin{array}{l} \text{Sparsity}=\frac{1}{m}N_{\text{imp}} \end{array}$$

where *m* is the number of neurons, and *N*_imp_ is the number of spikes emitted by the *m* neurons in response to an input vector over the time window *T*. A low value indicates a large number of inactive neurons and therefore a high sparsity.

#### 3.1.3 Coherence in the election of the SBMU

The Best Matching Unit (BMU) in traditional Vector Quantization models like the Self-Organizing Map (Kohonen, 2013), is selected each iteration using global network information. However, in our SNN, the Spiking Best Matching Unit (SBMU) selection is a dynamic and self-organized process influenced by neuron competition to represent the current input.

To assess the coherence of this self-organized SBMU selection compared to a Euclidean distance-based selection, we introduce a measure of incoherence. This measure evaluates if the SBMU is among the best representatives minimizing the Euclidean distance between the reconstructed patch from the SBMU and the input.

Firstly, we define a function *d* which, for a given input vector, calculates the normalized Euclidean distance between the input vector and the associated code vector of a neuron indexed *j* ∈ 1, …, *m* in the representation layer:


$$\begin{array}{l} d:1,\ldots ,m\to \left[0,1\right] \end{array}$$

Then, all distances *d*(*j*) are computed, and pairs [*j, d*(*j*)] for *j* = 1, …, *m* are sorted in increasing distance order, resulting in an ordered list [*j*_*p*_, *d*(*j*_*p*_)], where *p* = 1, …, *m*, and *j*_*p*_ denotes a pair index in the list:


$$\begin{array}{l} \forall p<m\;d\left(j_{p+1}\right)\geq d\left(j_{p}\right)\;\text{and }\left\{j_{p}|p=1,\cdots \;,m\right\}=\left\{1,\cdots \;,m\right\} \end{array}$$

Next, we assess if the index of the selected SBMU lies within the top *x*% of the ordered list. If yes, we increment the coherent SBMU count (sc), otherwise the paradoxical SBMU count (sp) is incremented. This check is repeated for each input vector to classify the elected SBMU as coherent or paradoxical. The top *x*% of the list sets the tolerance threshold for distinguishing between a coherent and paradoxical SBMU.


$$\begin{array}{l} \text{if sbmu}\in j_{p}|p\in 1,\ldots ,\left\lceil mx\right\rceil ,\;\text{then}sc\leftarrow sc+1\\ \text{else}sp\leftarrow sp+1 \end{array}$$

Lastly, the incoherence measure is defined, with sc and sp denoting the total number of coherent and paradoxical SBMU, respectively:


$$\begin{array}{l} \text{Incoherence}=1-\frac{sc}{sc+sp} \end{array}$$

High incoherence may for instance potentially lead to a high root mean square (RMS) reconstruction error.

### 3.2 Parameters of W-TCRL

A common challenge in training spiking neural networks (SNNs) is their limited adaptability to varying data dimensionalities, often requiring extensive hyperparameter tuning.

To address this issue, we propose a generic parametrization approach for the W-TCRL model that can handle arbitrary data dimensionalities, denoted as *k*. Only three parameters in the representation layer, namely the neuron firing threshold $V_{\theta }^{v}$, the initial lateral inhibition level *c*_min_, and the final inhibition level *c*_max_, depend on the data dimensionality. For simplicity, we assume a linear relationship between the data dimensionality *k* and these three parameters.

In our parametrization, each encoder neuron emits a single spike to represent a continuous input, and the total number of spikes in the encoding layer is proportional to the data dimensionality *k*, given by *k*×*l*, where *l* represents the number of neurons used to represent each dimension of the input space. As spikes are weighted by synaptic weights within the range of [0, 1], a neuron can only fire if its firing threshold $V_{\theta }^{v}$ is set to *c* × *k* × *l*, where *c* is a coefficient ranging from 0 to 1.

Additionally, to maintain a Winner-Take-All (WTA) behavior in the representation layer, it is crucial to calibrate the lateral inhibition level based on the data dimensionality. This ensures that the inhibition level is sufficiently high to prevent other neurons from reaching their firing threshold $V_{\theta }^{v}$.

We determined the coefficients of the linear equations relating these three parameters to the input data dimensionality *k* and the number of encoder neurons *l* using the Bayesian optimization method known as Tree-structured Parzen Estimator (TPE) (Bergstra et al., 2011). Our objective was to minimize the root mean square (RMS) reconstruction error on the MNIST and natural image datasets, considering input vectors with varying dimensions. The aim was to obtain generic and robust coefficients capable of handling diverse input data distributions and dimensionalities.

By incorporating the optimized coefficient values into the linear equations, the values of the three hyperparameters can be determined as follows:


(8) $$\begin{array}{ll} V_{\theta }^{v} & =0.25kl \end{array}$$

(9) $$\begin{array}{ll} c_{\text{min}} & =9V_{\theta }^{v} \end{array}$$

(10) $$\begin{array}{ll} c_{\text{max}} & =91V_{\theta }^{v} \end{array}$$

The parameters of W-TCRL used in all simulations are given in Table 1.

**Table 1:** W-TCRL parameters used in all simulations.

**Neuronal parameters**						
**dt**	$\tau_{m}^{u}$	$\tau_{m}^{v}$	$V_{\theta }^{u}$	$V_{\theta }^{v}$	$T_{\text{refrac}}^{u}$	$T_{\text{refrac}}^{v}$
0.1 ms	10.0 ms	1.4 ms	0.5	0.25 *kl*	6 ms	6 ms
**Synaptic parameters**						
**τ_*f*_** **(u** **to** **v)**	**τ_*f*_** **(v** **to** **v)**	**τ_*x*_**	**τ_*y*_**			
2.8 ms	0.3 ms	1.3 ms	4.3 ms			
**STDP rule parameters**						
**α^+^**	**α^−^**	***w*_offset_**	**ϵ**			
0.004	0.024	0.2	0.1			
**Homeostatic mechanism parameters**						
**τ_*w*_**	***c*_min_**	***c*_max_**				
1/3 *TP*	9 $V_{\theta }^{v}$	91 $V_{\theta }^{v}$				

### 3.3 Results for MNIST

Experiments were run with varying network sizes with *m* ∈ {16, 32, 64, 128, 256} neurons in the representation layer and therefore *m* code vectors submitted to learning. Each experiment was evaluated with 30 independent simulations. For a fair comparison with the state of the art set by Tavanaei et al. (2018), we used the same experimental protocol, including the lack of cross-validation.

Synaptic weights between the encoding layer *u* and the representation layer *v* are randomly initialized in the interval [0.6, 0.8]. The inputs are normalized within the interval [0.15, 0.85]. We introduce a margin due to the projection of linear input data onto a circular space (receptive fields) where extreme values become equivalent (2 π is equivalent to 0 radians). In addition, MNIST is essentially composed of extreme values (black and white pixels).

For training and testing, subsets of 15,000 and 1,000 handwritten digits were respectively used. These subsets provided 5 × 5 pixel patches extracted from 28 × 28 pixel digits as SNN inputs. The dimension-dependent parameters $V_{\theta }^{v}$, *c*_min_, *c*_max_ were automatically determined for 5 × 5 = 25 dimensions by Equations (8), (9), (10).

In the training phase, 60,000 image patches were utilized, while the test phase held the lateral inhibition in the representation layer at its maximum (*c*_max_), with plasticity disabled.

Performance was assessed based on the three aforementioned metrics: the RMS reconstruction error between an input vector and the code vector of the SBMU, the sparsity of the representation layer activity, and the incoherence in the self-organized process of SBMU election.

#### 3.3.1 Learning phase

**Figure 6:**
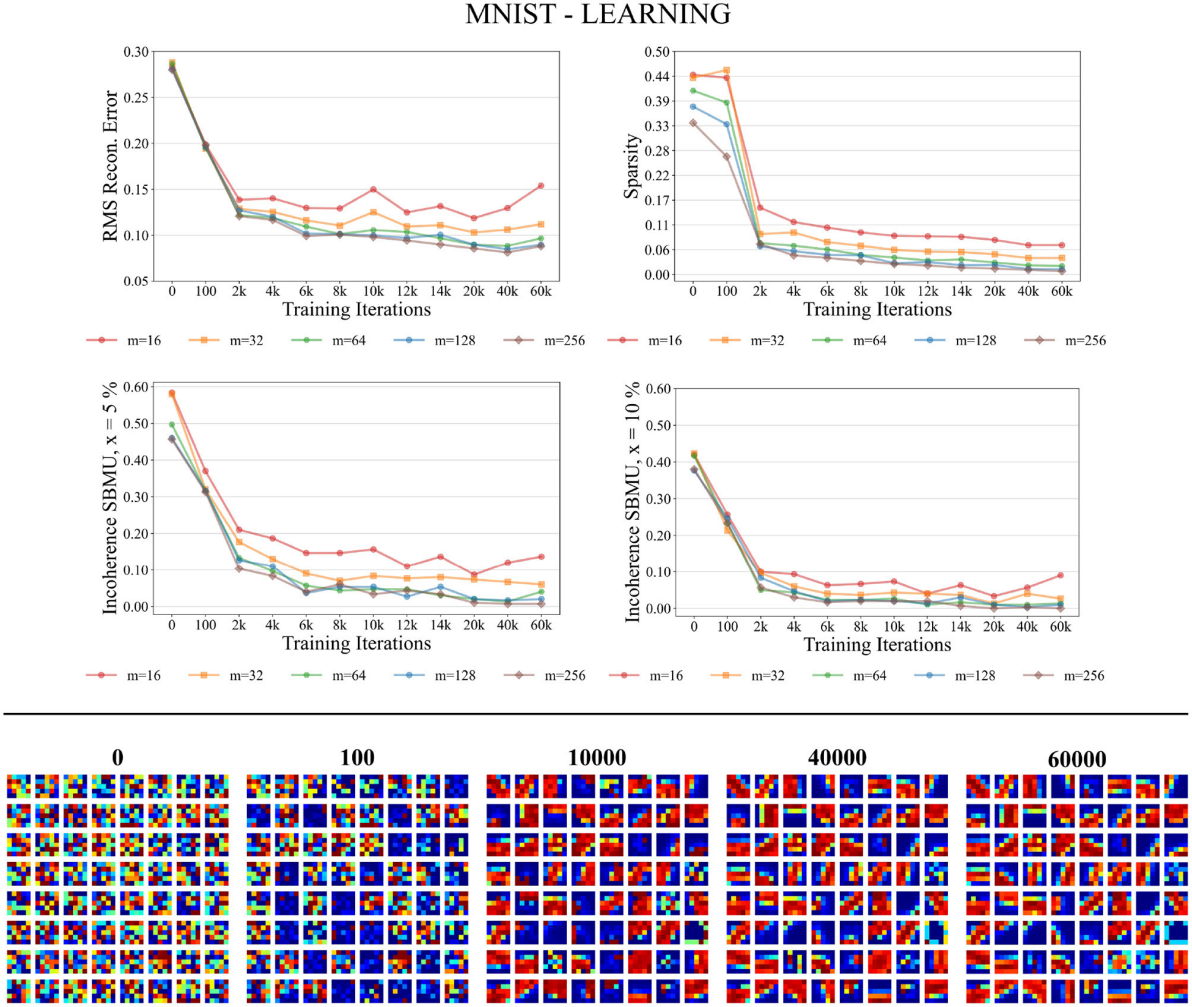
Performance for MNIST during the training phase. **Top**: Average model performance for different network size through training iterations in terms of RMS reconstruction error, sparsity, and incoherence for a decision threshold *x* at 5 and 10%. **Bottom**: Decoded code vectors for *m* = 64 neurons after 0, 100, …, 60,000 training iterations. The blue-red gradient represent minimum and maximum values, respectively.

Figure 6 shows that the different performance measures consistently enhance with the progression of learning iterations, and also with an increasing number of neurons *m* ∈ {16, 32, 64, 128, 256} in the representation layer *v*. The RMS reconstruction error decreases, indicating that W-TCRL learns to compress the distribution of input data, reaching a relatively stable plateau after 8,000 iterations with an RMS error of about 0.08 for *m* ∈ {64, 128, 256} neurons. The sparsity also decreases, indicating that the percentage of neurons firing in the representation layer *v* decreases in response to an input vector. This is attributed to heightened lateral inhibition through homeostasis and increased neuronal specialization. Lastly, Euclidean incoherence in the SBMU election process reduces through training iterations for a decision threshold *x* at 5 and 10%, signaling fewer paradoxical SBMUs and improved neuronal receptive field selectivity for specific input regions. This shows that the first firing neuron is, on average, an accurate representative of the input spike pattern.

Figure 6 shows that the code vectors in the representation layer gradually become selective to various visual orientations over the course of the learning iterations.

#### 3.3.2 Testing phase

**Figure 7:**
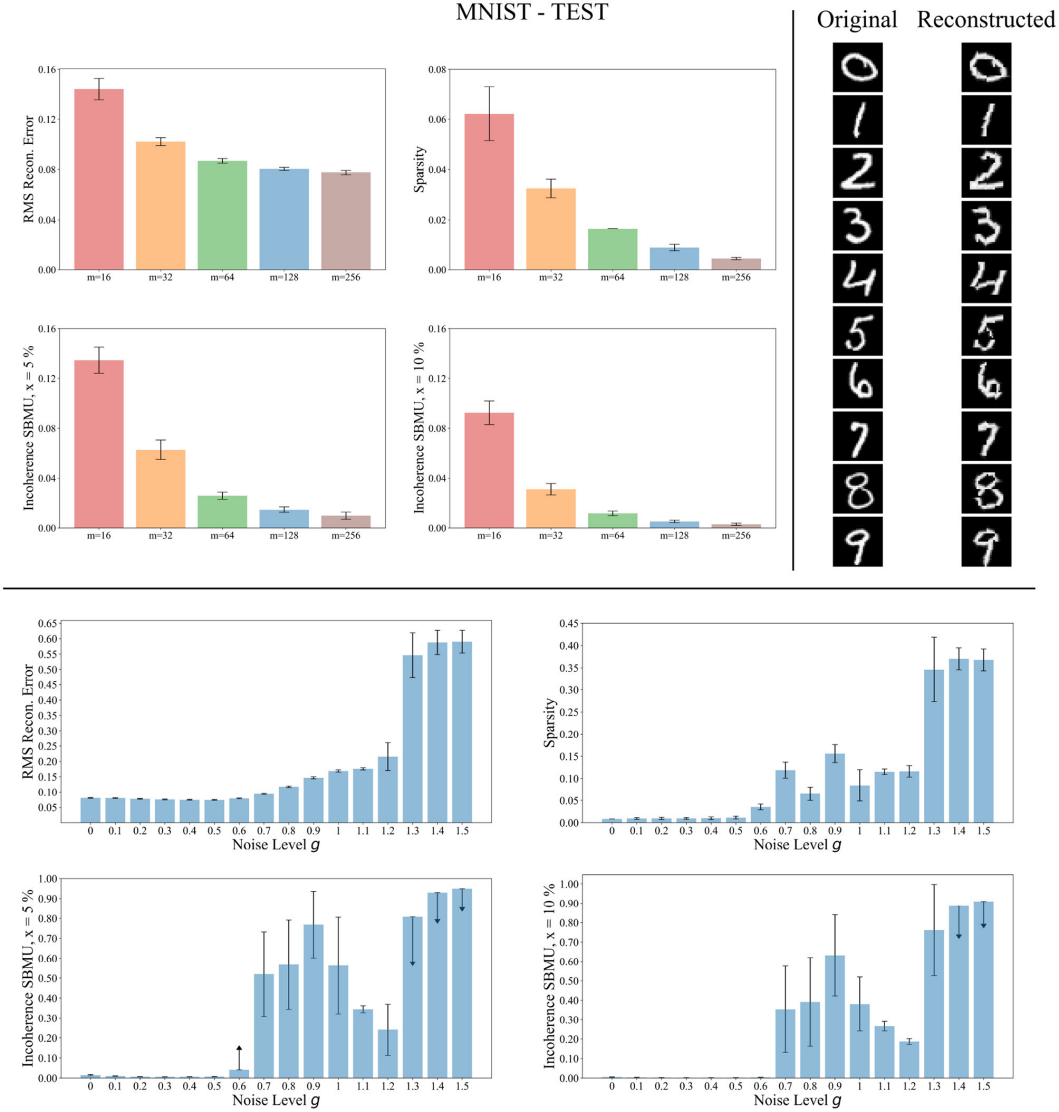
Performance for MNIST during the testing phase. **Top left**: Average model performance for different network size in terms of RMS reconstruction error, sparsity and incoherence for a decision threshold *x* at 5 % and 10 %. Error bars show standard error of the mean for 30 independent runs. **Top right**: Set of original images presented as input to the SNN and reconstructed using the code vectors of neurons in the representation layer, with *m* = 128 neurons. **Bottom**: Average model performance for different noise levels *g* with *m* = 128 neurons. Error bars show standard error of the mean for 30 independent runs.

We now evaluate the performance of W-TCRL on the test dataset for MNIST. The achieved performances all increase with the capacity of the SNN (see Figure 7). The average sparsity for *m* = 256 neurons reaches a value of 0.004, meaning that only 0.4% of the neurons in the representation layer are active on average. Self-organized SBMU selection is achieved with up to 99.0% of coherent SBMU for a decision threshold of 5% and *m* = 256 neurons. The best RMS reconstruction error reaches the value of 0.08 (see Table 2). This represents a relative improvement of 53% compared to the SNN state-of-the-art (see Table 3) for learning representations using the MNIST dataset.

**Table 2:** Average results on 30 independent runs obtained on MNIST and natural images datasets for a given number of neurons *m* ∈ {16, 32, 64, 128, 256}.

**Dataset**	**MNIST**					**Natural images**				
**Neurons m**	**16**	**32**	**64**	**128**	**256**	**16**	**32**	**64**	**128**	**256**
RMS	0.144	0.102	0.087	0.080	0.078	0.164	0.138	0.061	0.056	0.056
Sparsity	0.062	0.032	0.016	0.009	0.004	0.255	0.234	0.141	0.076	0.043
Incoherence 5 %	0.134	0.063	0.026	0.015	0.010	0.516	0.473	0.361	0.275	0.224
Incoherence 10 %	0.092	0.031	0.012	0.005	0.003	0.378	0.344	0.271	0.176	0.064

**Table 3:** Best RMS reconstruction errors reported by Tavanaei et al. (2018) are compared to our model W-TCRL.

**Dataset**	**Tavanaei**	**W-TCRL (our model)**
MNIST	0.17	**0.08**
Natural images	0.24	**0.06**

Bold values represent the performances of our model.

The reconstruction of images from the self-organized elected code vectors found by W-TCRL produces well-reconstructed handwritten digits, comparable to the original images (see Figure 7). The quality of the reconstructed digits demonstrates the effectiveness of our SNN architecture in capturing and representing the visual information accurately.

We also tested the robustness of our network's ability to learn under noise (see Figure 7). During the training phase, we varied the white noise scaling factor *g* in the range [0, 1.5], resulting in spike jitter up to around 5 ms in spikes outputted by the encoding layer. The performance remains consistent across the different metrics for values of *g* up to 0.5, demonstrating that noise doesn't prevent neurons in the representation layer from learning statistical correlations from input spikes. However, with *g*>1.2, the noise becomes too strong. It destroys the temporal information contained in the spike pattern. Consequently, the neurons fail to extract meaningful representations (high RMS reconstruction error), tend to fire simultaneously (low sparsity), and lose their selectivity (high incoherence).

### 3.4 Results for natural images

As for MNIST, experiments for the natural images dataset were conducted for several network sizes with *m* ∈ {16, 32, 64, 128, 256} neurons in the representation layer and thus *m* code vectors subject to learning. Each experiment was evaluated with 30 independent simulations.

The synaptic weights between the encoding layer *u* and the representation layer *v* are randomly initialized in the interval [0.6, 0.8]. Input normalization was performed to scale inputs within the interval [0.05, 0.95]. 16 × 16 pixel patches, extracted from natural images of 512 × 512 pixels, are provided as input vectors to the SNN. The dimension-dependent parameters $V_{\theta }^{v}$, *c*_min_, *c*_max_ were automatically determined for 16 × 16 = 256 dimensions using Equations (8), (9), (10). A total of 60,000 image patches are provided to the SNN during the training phase. In the test phase, lateral inhibition in the representation layer *v* was set to its maximum value, *c*_max_, and plasticity was disabled.

Similarly to the previous section, we evaluated the performance of W-TCRL using the same three metrics: the root mean squared (RMS) reconstruction error, the sparsity of the representation layer activity, and the Euclidean incoherence of the SBMU.

#### 3.4.1 Training phase

**Figure 8:**
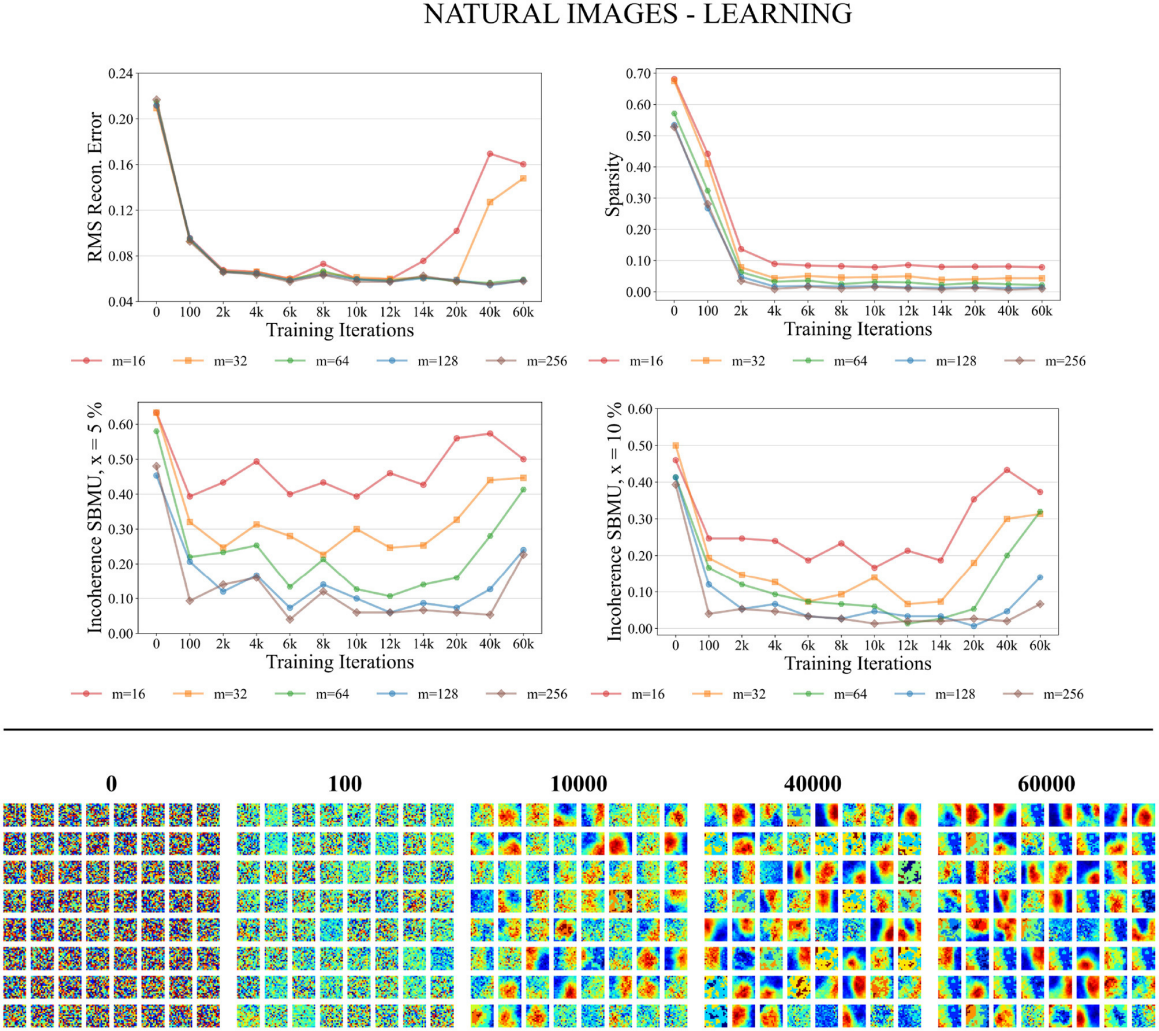
Performance for natural images during the training phase. **Top**: Average model performance for different network size through training iterations in terms of RMS reconstruction error, sparsity, and incoherence for a decision threshold *x* at 5 and 10%. **Bottom**: Decoded code vectors for *m* = 64 neurons after 0, 100, …, 60,000 training iterations. The blue-red gradient represent minimum and maximum values, respectively.

As depicted in Figure 8 the performance measures for the natural images dataset in the training process consistently improve as the number of neurons in the representation layer increases. When the network capacity is too small, specifically with 16 and 32 neurons in the representation layer, the RMS reconstruction error is significantly degraded. In the case of higher network capacities, specifically with 64, 128, and 256 neurons in the representation layer, the RMS reconstruction error exhibits a consistent decrease throughout the training process. After approximately 6,000 iterations, the RMS error reaches a stable plateau, converging to a value of approximately 0.06. The sparsity of the representation layer activity decreases over the course of training, reaching a plateau after 6,000 iterations. The euclidian incoherence of the SBMU election decreases when using a decision threshold of 5% and 10%, but the euclidian incoherence exhibits more fluctuations compared to the results obtained for the MNIST dataset. This observation can be attributed to the high dimensionality of the input data (256 dimensions), which causes the Euclidean distances to become concentrated and thus difficult to distinguish. This hypothesis is further supported by a low RMS reconstruction error and its low fluctuation.

Figure 8 demonstrates that the code vectors in the representation layer exhibit an increasing selectivity toward visual patterns as the number of training iterations increases.

#### 3.4.2 Testing phase

**Figure 9:**
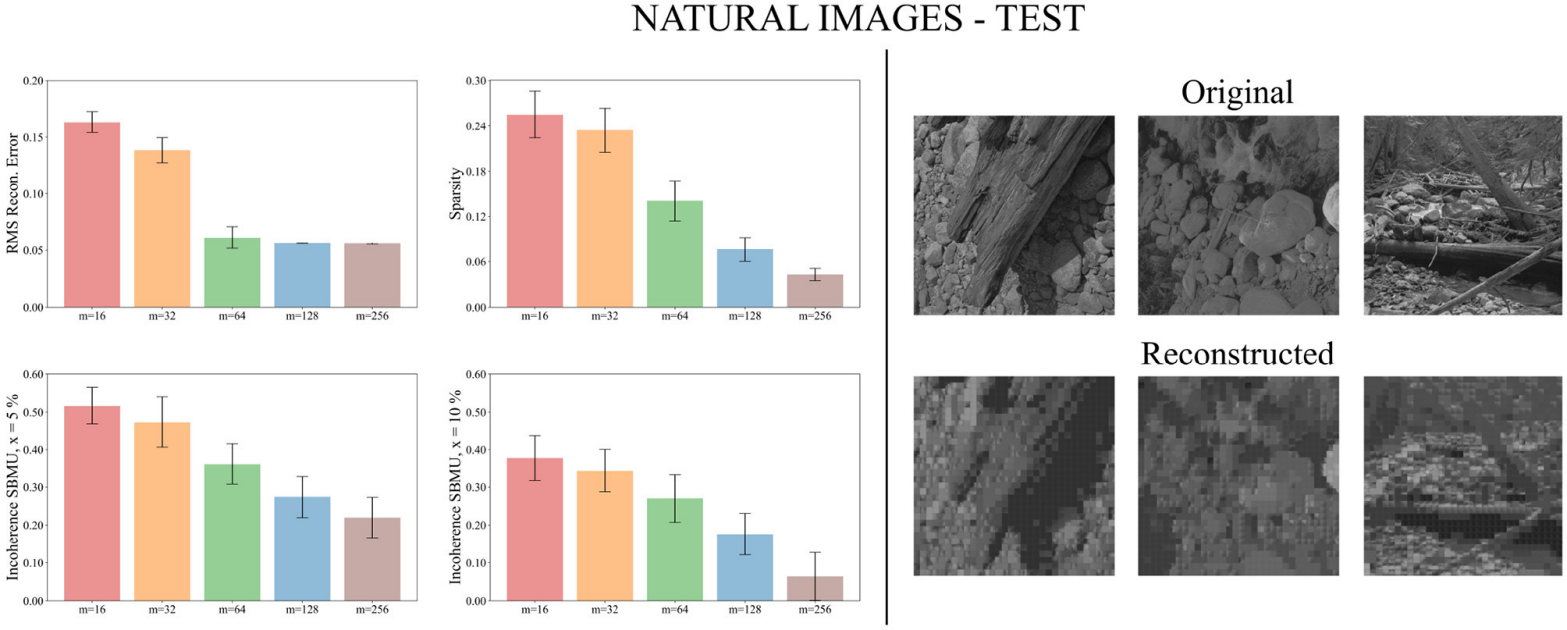
Performance for natural images during the testing phase. **Left**: Average model performance for different network size in terms of RMS reconstruction error, sparsity and incoherence for a decision threshold *x* at 5 and 10%. Error bars show standard error of the mean for 30 independent runs. **Right**: Set of original images presented as input to the SNN and reconstructed using the code vectors of neurons in the representation layer, with *m* = 128 neurons.

We now evaluate the performance of W-TCRL on the test dataset for natural images. The obtained performance increases as the capacity of the SNN increases, i.e., the number of neurons *m* ∈ {16, 32, 64, 128, 256} in the representation layer (see Figure 9). The average sparsity for *m* = 256 neurons reaches a value of 0.04 indicating that only 4% of the neurons in the representation layer are active on average. The self-organized election of the SBMU is achieved with up to 95.6% of coherent SBMU for a decision threshold of 10% and *m* = 256 neurons. The RMS reconstruction error reaches a plateau at 0.06 for *m* ∈ {64, 128, 256} neurons (see Table 2). This represents a relative improvement of 75% compared to the SNN state of the art (see Table 3) for this dataset of natural images.

Figure 9 demonstrates that the self-organized elected code vectors of W-TCRL yield reconstructed images that closely resemble the original images (though the visual quality inherently suffers from large sizes of patches, regardless of the model used for representation learning).

## 4 Discussion

In contrast to rate-based encoding methods, which require up to 255 spikes for an 8-bit pixel value (King et al., 2013; Burbank, 2015; Tavanaei et al., 2018), our approach employs latency-based encoding with population coding (Ebitz and Hayden, 2021). This method uses 10 neurons per input coordinate, each emitting a single spike, resulting in a sparse and efficient encoding with only 10 spikes.

Our encoding method diverges from the widely used Time-To-First-Spike (TTFS) coding, which necessitates knowledge of stimulus onset timing. This information is available to the experimenter but not the neurons, which makes it biologically implausible. In contrast, our use of population coding embeds information within the spike pattern itself, specifically in the timing relative to other neurons in the population. This allows downstream neurons to process these spike patterns in an event-based manner. More generally, population-based latency coding can be viewed as an extension of TTFS. It provides a more flexible representation by distributing information across multiple dimensions (population of neurons) rather than one (single neuron). This enhances flexibility and transmission speed with minimal extra spikes. Although population-based latency coding does not require an external time reference, it does need an internal one, similar to rate coding which needs a temporal window for averaging spikes. As Zhou et al. (2012) noted, the brain may generate several internal time references, including large-scale oscillations (Hopfield, 1995), local field potentials (Eggermont, 1998), and population onsets (Stecker and Middlebrooks, 2003; Chase and Young, 2007).

One limitation of latency-coding is its sensitivity to noise (Guo et al., 2021). This issue is not exclusive to this coding scheme. All neural codes relying on precise spike timing are vulnerable to noise and jitter. A potential solution is to pool information from a neuron population, as we have done, instead of relying on a single neuron as in TTFS. Our experimental results demonstrate that the combination of population-based latency coding with our new STDP rule makes the model quite robust in learning from noisy input spike patterns. The correlation among a neuron population can establish an error-correcting code that mitigates noise effects. This is because the spike timing jitter in relation to other neurons could be less than the jitter referenced to the onset of the stimulus (Zhou et al., 2012).

The extraction of representations from a temporal code lacked event-based local learning rules aimed at achieving low reconstruction error. Our work bridges this gap by introducing a novel STDP rule that extracts spatio-temporal centroids. This simple rule enables stable learning of the distribution of relative latencies within the synaptic weight distribution. Unlike backpropagation algorithms, which require costly global information transport and significant memory for sequence history storage, our STDP rule operates on an event-based and local level in both time and space. The event-based and local operations of our STDP rule align well with the principles of asynchrony and locality inherent in neuromorphic processors (Davies et al., 2018). This makes our STDP rule an ideal fit for such implementations.

Early presynaptic spikes, carrying the most input information, trigger the first (causal) case of weight adaptation on postsynaptic spikes, leading to synaptic weights *w*_*ji*_>0. The learned weight *w*_*ji*_ is a function of the latency Δ_*t*_ = *t*_*post*_−*t*_*pre*_ between the pre and postsynaptic spikes. Earlier spikes result in higher weights and have a greater impact on postsynaptic spike emission. This differs from the classical STDP rule, which maximizes weight strengthening for late-arriving spikes (*t*_*pre*_≈*t*_*post*_) and usually leads to weight saturation rather than converging to a stable value. Later presynaptic spikes, containing less information, trigger the second (anti-causal) case of weight adaptation, causing synaptic depression (*w*_*ji*_ → 0). Through these adaptations, a postsynaptic neuron learns a code vector or filter in its afferent synaptic weights. This facilitates rapid decision-making before receiving all presynaptic spikes, thereby accelerating the SNN's processing speed and mirroring biological neurons.

Rank-order TTFS coding (Thorpe and Gautrais, 1998) follow a similar approach to build detectors, where the weights decrease linearly with the spike rank. The synaptic weights are M (maximum) for the first spike, then M-1, …,1. This approach focuses on the spike order rather than their precise timings, contrary to the basis of our approach. An accompanying modulation function emphasizes the importance of earliest spikes. The conjunction of a set of weights and a modulation function allows to build accurate detectors of spike patterns (Bonilla et al., 2022). This modulation function concept can be related to other TTFS coding works that utilize precise spike timing for supervised learning tasks (Rueckauer and Liu, 2018; Zhang et al., 2022; Sakemi et al., 2023). These studies enhance the impact of the earliest spike by linearly increasing the postsynaptic potential (PSP) over time. Our model naturally achieves a similar effect through the CuBa LIF model. The PSP shape of the CuBa LIF is determined by the ratio τ_*f*_/τ_*m*_ of the synapse's exponential decay time constant to that of the neuron (Göltz et al., 2021). By selecting τ_*f*_>τ_*m*_ as done in our experiments, the earliest spikes have a greater impact on the postsynaptic neuron response due to their influence being integrated over a longer period.

To implement competitive learning among neurons in the representation layer, our SNN incorporates lateral inhibitory connections. During the learning phase, a mechanism is employed to initially recruit multiple neurons, which accelerates the convergence of the SNN. Subsequently, the number of recruited neurons gradually decreases to promote specialization and decorrelation of the learned code vectors. This behavior is achieved through a homeostatic mechanism that increases the magnitudes of inhibitory lateral weights. As a result, the circuit transitions dynamically from a k-winners-take-all (k-WTA) to a winner-take-all (WTA) behavior, thereby increasing the sparsity of activity in the representation layer.

While representation learning in ANNs is a mature field, achieving good performance with SNNs remains challenging. Our W-TCRL model, based on temporal coding and a novel STDP rule, outperforms the state-of-the-art of SNNs using a rate-based coding approach by Tavanaei et al. (2018) in terms of RMS reconstruction error. Our method exhibits a relative improvement of 53% for MNIST and 75% for natural images. The reconstructed images exhibit a good visual quality compared to the original images.

The representation layer exhibits a highly sparse activity during inference, a desirable feature (Frenkel, 2021). For instance, on the MNIST dataset with *m* = 256 neurons, the average sparsity is 0.004, indicating that only 0.4% of neurons in the representation layer fire. Notably, neurons in the representation layer fire only once, distinguishing our approach from rate coding methods. In the work of Tavanaei et al. (2018), sparsity was averaged over a time window of *T* = 40 time steps for a given number of neurons *m*. The reported best sparsity of 9% implies that, on average, 9% of neurons fire at each time step within the encoding window *T*. In contrast, in our temporal coding-based SNN, a sparsity of 0.4% means that 0.4% of neurons fire a single time during the encoding window *T*. Therefore, based on the best reported sparsity by Tavanaei et al. (2018), a neuron fires an average of 0.09*40 = 3.6 spikes during the temporal window *T*. In contrast, based on our best sparsity results, a neuron emits an average of 0.004 spikes during the temporal window *T*. Thus, our method achieves up to 900 times fewer spikes emitted per neuron in the representation layer on average, providing substantial benefits in terms of energy consumption and bandwidth in spike communication protocols such as AER, while improving the RMS reconstruction error. This empirical evidence confirms the benefits of using temporal codes compared to rate coding.

Lastly, the self-organized selection of the Spiking Best Matching Unit (SBMU) in our SNN achieves up to 99.0% coherent SBMUs (for MNIST with *m* = 256 neurons and a decision threshold *x* set to 5%). The high coherence of the SBMUs indicates that the neurons in the representation layer have developed a high level of selectivity for specific regions within the spatio-temporal input space. This selectivity results in an effective clustering of the spatio-temporal input space.

Future research should prioritize implementing our spiking representation learning model on neuromorphic processors (Davies et al., 2018) to enable real-time, low-power, and high-throughput processing, taking advantage of their parallelism and efficiency. Additionally, exploring the unsupervised extraction of hierarchical representations from sensory data by stacking multiple layers using our STDP rule can uncover more complex and abstract features in self-organizing SNN architectures. This unsupervised approach is exemplified by the HOTS architecture (Lagorce et al., 2017), which employs a three-layer stack for unsupervised feature extraction, with the final layer's output fed to a simple histogram classifier that achieves near 100 % accuracy on DVS datasets.

## Data availability statement

Publicly available datasets were analyzed in this study. These data can be found at: MNIST: http://yann.lecun.com/exdb/mnist/; Natural Images: http://www.rctn.org/bruno/sparsenet/.

## Author contributions

AF was the originator of the W-TCRL model. AF and BG developed its formalized expression and its analysis. AF carried out all simulations, and wrote the manuscript with contributions of BG. Both authors contributed to the article and approved the submitted version.

## Footnotes

^1^The code is released: https://github.com/afois/W-TCRL.
